# Investigating thermal stability based on the structural changes of lactase enzyme by several orthogonal methods

**DOI:** 10.1016/j.btre.2021.e00637

**Published:** 2021-05-26

**Authors:** Márton Király, Borbála Dalmadi Kiss, Péter Horváth, László Drahos, Arash Mirzahosseini, Gyula Pálfy, István Antal, Krisztina Ludányi

**Affiliations:** a Department of Pharmaceutics, Faculty of Pharmacy, Semmelweis University, Hőgyes Endre u. 7., 1092, Budapest, Hungary; b Department of Pharmaceutical Chemistry, Faculty of Pharmacy, Semmelweis University, Hőgyes Endre u. 7., 1092, Budapest, Hungary; c MS Proteomics Research Group, Research Centre for Natural Sciences, Magyar Tudósok körútja 2., H-1117, Budapest, Hungary; d Laboratory of Structural Chemistry and Biology, Institute of Chemistry, Eötvös Loránd University, Pázmány P. sétány 1/A, 1117, Budapest, Hungary; e Protein Modeling Group HAS-ELTE, Institute of Chemistry, Eötvös Loránd University, 1538, Budapest, P.O.B. 32, Hungary

**Keywords:** NMR, Proteomics, β-Galactosidase, Aggregation, Mass spectrometry

## Abstract

Thermal stability of lactase (β-galactosidase) enzyme has been studied by a variety of physico-chemical methods. β-galactosidase is the main active ingredient of medications for lactose intolerance. It is typically produced industrially by the Aspergillus oryzae filamentous fungus. Lactase was used as a model to help understand thermal stability of enzyme-type biopharmaceuticals. Enzyme activity (hydrolyzation of lactose) of β-galactosidase was determined after storing the solid enzyme substance at various temperatures. For a better understanding of the relationship between structure and activity changes we determined the mass and size of the molecules with gel electrophoresis and dynamic light scattering and detected aggregation processes. A bottom-up proteomic procedure was used to determine the primary amino acid sequence and to investigate changes in the N-glycosylation pattern of the protein. NMR and CD spectroscopic methods were used to observe changes in higher order structures and to reveal relationships between structural and functional changes.

## Highlights

• The primary and secondary structure of lactase was not changed by thermal stress.
• The glycan structure of lactase showed significant alterations after heat stress.
• Changes in the glycosylation structure affected physico-chemical properties of lactase.

## 1 Introduction

Biologicals produced by biotechnology are becoming widely used. Quality assurance and stability testing of these complex protein type molecules demand extensive knowledge of their structure in order to provide effective and safe medications. Stability testing of immunoglobulin-based biologicals is relatively well established, but for other biologicals, like large, enzyme type drugs it has not yet been defined. Stability and quality assessments are usually based on simple activity tests, without any information on possible changes in the structure or in the posttranslational modifications of the enzyme. Stress measurements of the enzyme are usually performed in the form of a solution, although the formulations and technological processes are mainly present in the solid form.

β-galactosidase, also called lactase is a hydrolytic enzyme that decomposes lactose, a disaccharide found in dairy products. It also catalyses the reverse of the reaction, called trans glycosylation [1]. β-Galactosidases of various origins belong to families of glycosyl hydrolases produced by almost every living organism. The lack or decreased production of this digestive enzyme in the human small intestine results in the inability to properly break down and assimilate milk containing products. These dairy products play an important role in gastronomy in many parts of the world, irregularity of its metabolism (insufficient hydrolysis of lactose) may result in a serious deterioration of the quality of life [2,3]. The prevalence of the lactose intolerance is over 60 % amongst the human population, causing health problems of varying severity [4]. The treatment of this disease involves the administration of various medications containing lactase. These pharmaceutical products: tablets, chewing-tablets, capsules, solutions etc. usually contain one specific stream of lactase derived from Aspergillus oryzae (Yellow koji mold), also called Tilactase [5]. The beta-galactosidase produced by this medically and industrially important filamentous fungus is a relatively big and complex glycoprotein, which contains 1009 amino acids, and has an estimated molecular mass of 109 kDa without the post translational modifications [6].

Enzyme molecules like lactase have many different structural variants with different properties and resistance to external effects (pH, temperature etc.), depending on its source. [7]. Structural differences behind this phenomenon have not been explored yet, but it seems likely that the glycan structures play an important role in the stability and efficiency of the protein [[8], [9], [10], [11]]. Enzyme type active ingredients are usually sensitive to pH, moisture, pressure, temperature, applied excipients and reactants (metal ions) occurring during manufacture. There is a possibility that they may lose their effectiveness before or during oral administration, although various technologies try to overcome these disadvantages [[12], [13], [14], [15]]. Forced degradation studies are often performed on active pharmaceutical ingredients to determine and predict possible degradation pathways under various stress conditions. A large variety of degradation processes may result in the loss of function or loss of efficiency, and these are very complex to analyse [16]. Currently, there are no exact [17] guidelines for the industry defining how to perform stability testing in case of protein type active ingredients. [18].

Despite the pharmaceutical and commercial relevance of the lactase containing products [19], our knowledge is limited about the relationship between their structure and activity. In the present study we will use a combination of analytical tools in order to discover structural changes caused by stress conditions, which influence enzyme activity and function. The lactase enzyme in pulverized form will be studied (i.e., in the solid form), which is typical in and production storage. Bottom-up proteomic procedure utilizing nanoUHPLC-MS method was used to observe the possible alterations in the primary amino acid sequence and changes in PTMs, such as N-glycan structures. Beside mass spectrometric measurements SDS-PAGE, DLS, NMR, CD methods were applied to observe the possible changes in the primary and in the higher ordered structures and to look for possible aggregation. NMR and CD techniques were used to determine alterations in the secondary and tertiary structures. Parallel ortho-nitrophenyl-β-galactoside (ONPG) based activity studies have been performed to establish structure-activity relationship.

The main objective of the research is to reveal structural changes of the lactase enzyme under heat-stress conditions. This is important both for understanding fundamental aspects of enzyme activity changes under storage, and for practical considerations such as improving dosing, manufacturing, shipping and storage of biopharmaceuticals, like lactase containing products [20].

## 2 Materials and methods

### 2.1 Materials

The powder of β-galactosidase enzyme (Opti-lactase A-50 powder) derived from Aspergillus oryzae was purchased from Optiferm Gmbh (Oy-Mittleberg, Germany). O-nitrophenyl-d-galactopyranoside (ONPG), MU-Gal (4-Methylumbelliferyl β-d-galactopyranoside) and the broad range SDS–PAGE molecular weight standards were obtained from VWR International (Radnor, PA, US). For the tryptic digestion Trypsin Gold, Mass Spectrometry Grade (Promega Corporation, Madison, WI, US) and Rapigest (Waters, Milford, MA, US) were used. Ultrapure water obtained from the MilliRO-hMilliQ-system (Merck Millipore, Burlington, MA, US) was used throughout all measurements. All other ingredients like the reagents, solvents, eluents were obtained from Sigma Chemical Company (St. Louis, MO, US).

### 2.2 Stress stability tests

The essence of the stress stability test is to model the behaviour of the sample under extreme conditions, such as high temperatures to determine and model extreme storage and production parameters [21], and predict processes occurring among normal conditions. During the temperature-induced stress test the native protein powder samples (1000 mg each) were incubated at several temperatures for different time periods and were kept in locked glass vials. The heat load took place in a stability test chamber kept and controlled at a constant temperature and humidity. Two control samples were stored at room temperature and refrigerated. The exact experimental conditions are summarised in Table 1.

**Table 1:** Experiment layout of thermal stability stress tests.

Conditions	Storage Time				
40 °C	1 day	3 days	5 days	1 week	2 weeks
60 °C	1 day	3 days	5 days	1 week	2 weeks
80 °C	1 day	3 days	5 days	1 week	
Control 1 (25 °C room temp.)	1 day				2 weeks
Control 2 (5−8 °C refrigerated)	1 day				2 weeks
−20 °C deep frozen	1 day				2 weeks

### 2.3 Activity measurement

The hydrolysing activity of the examined enzyme stored at various temperatures for various time periods listed above was estimated using o-nitrophenyl-β-d-galactopyranoside (ONPG) as substrate [22]. ONPG is a colourless substance, which is cleaved by β-galactosidase to galactose and o-nitrophenol (ONP), the reaction is indicated by a yellow colour change. The reagent was solved in pH = 4.5 phosphate-citrate (McIlvaine) buffer solution [23]. 5 ng/mL concentration samples were made with MilliQ water and the two solutions were mixed in a ratio of 1:3 [24,25]. The samples were incubated in water bath at 37 °C for 30 min. Aliquots were withdrawn from the reaction mixture and pipetted directly into 1 mL Na_2_CO_3_ solution to stop the enzymatic hydrolysis and to produce the maximum possible absorption due to the liberated o-nitrophenolate ion. The solution was left to cool to room temperature and the absorbance of o-nitrophenol was determined by UV–vis spectroscopy using an ATi Unicam UV2 UV/VIS Spectrometer (UNICAM, Budapest, Hungary) at 420 nm in three parallel measurements with five different stressed samples.

### 2.4 DLS

The average diameter of the proteins in the different samples was determined by dynamic light scattering (DLS) using Zetasizer NanoZS™ (Malvern Panalytical Ltd., Malvern, UK). Absorbances of a sample have been investigated before each particle sizing by single beam UV–vis spectrophotometer, (8453 type; Agilent Technologies, USA) at λ max =633 nm, to determine the correct amount of sample scatterings vs. absorbances. Samples were illuminated by a HeNe laser (wavelength of 632.8 nm, 4.0 mW), an avalanche photodiode (APD) detection angle was 173° (backscatter mode). Protein samples were dissolved in MilliQ water in the concentration of 1 mg/mL to reduce nonlinearity effects on measurements by increased viscosity of solvent with higher concentrations. Measurements were made performing 15 runs of 10 s each, an equilibration time of at least 5 min was set before the measurement started. Size measurements were made in triplicate and were used immediately after preparation. The experiments were carried out at a constant room temperature of 25 °C. All solutions were analysed without prior filtration to assess if aggregates were present up to 10,000 nm (detection limit). The diameter of the solved molecules is related to the properties of the solvent, so refractive index and viscosity were tested. The refractive index was measured by a clinical refractometer, the viscosity by a Brookfiled Ametek DVE viscometer (Middleboro, MA, US) and it proved to be 1.031 and 0.8991 cP.

### 2.5 Electrophoretic analysis

SDS-PAGE was carried out using a 10 % Tris-Glycin gel and a vertical Novex Minigel electrophoretic system. 100 μL of 1 mg/mL lactase solution was mixed to 100 μL SDS sample buffer (62.5 mM Tris–HCl, 2 % SDS, 25 % glycerol, 0.01 % bromophenol blue, 5 % mercapto-ethanol or 100 mM DTT, pH = 6.8) denatured at 80 °C for 5 min. 15 μL of each sample was used and the electrophoresis was carried out at 35 mA and 150 V for 90 min until the bromophenol blue has reached the lower edges of the gel. Protein bands were visualised by staining with Coomassie Brilliant Blue R-250 in 50 % methanol, and 10 % acetic acid staining solution, distained by a solution of 5% methanol and 10 % acetic acid for overnight. Broad-range SDS–PAGE molecular weight standards purchased from VWR were used as molecular mass standards (Myosin 200 kDa, β-Galactosidase 120 kDa, Bovine Serum Albumine 91 kDa, Glutamic dehydrogenase 62 kDa, Ovalbumin 47 kDa, Carbonic Anhydrase 37 kDa, Myoglobin 28 kDa, Lysozyme 19 kDa, Aprotinin 9 kDa).

For the Zymography (correlation between activity and protein position) the gel is subjected to fluorometric analysis using MU-Gal (4-Methylumbelliferyl β-d-galactopyranoside) to test for β-galactosidase activity of the separated bands. This gel is soaked for 15 min in 100 mM sodium acetate buffer, pH = 5.0 with lateral shaking, 100 rpm. Then, it is incubated with the MU-Gal solution at 37 °C for 15 min. The zymogram gel is analyzed under UV light, λ = 365 nm to detect enzyme activity due to the release of 4-methylumbelliferone. This step should be performed quickly, as the fluorescent product diffuses within the gel, obscuring the bands.

Note that while SDS and reducing agent are used in the SDS-PAGE gel and running buffer, non-denaturing electrophoresis was carried out with the omission of SDS from the gel running and loading buffers, and the sample was pre-treated only under moderate temperature.

### 2.6 NMR

Data were acquired from two samples with two different NMR methods: 1D ^1^H and 2D diffusion-ordered spectroscopy (DOSY) NMR. The samples were: lactase standard 100 mg solved in MilliQ water, 10 % D_2_O, 1% DSS (standard) added, pH = 7.45. Heated lactase at 80 °C for 14 days 100 mg solved in MilliQ water, 10 % D_2_O, 1% DSS (standard) added, pH = 6.96. Before the NMR measurements the samples were filtered five times with Eppendorf centrifuge tube equipped with a membrane of 10 kDa cut. After the filtration, the samples were diluted to 500 μL with MilliQ Water then measured immediately. NMR measurements were performed on a Bruker Avance III 700 MHz spectrometer equipped with a 5 mm Prodigy TCI H&F-C/N-D, z-gradient probe head operating at 700.05 MHz for ^1^H. All spectra were processed with Bruker TOPSPIN software. ^1^H NMR measurements DOSY experiments were performed at 14 °C in accordance with a previous NMR study which compares diffusion constants with the size of proteins [26], using the stebpgp1s19 pulse sequence with water suppression for diffusion measurements. The lengths of diffusion delays and pulses were optimized to *δ* =5 ms, *Δ* = 300 ms. The strength of the diffusion gradient was linearly incremented in 32 equal steps, varying between 5 % and 95 % of its maximum value; the applied maximum gradient strength was 45.4 G/cm. The number of scans was adjusted for each sample to obtain reliable S/N ratios (16 for ^1^H and 40 or 160 for DOSY experiments). Each measurement was repeated at least three times. To investigate the diffusion constants signals were chosen from several regions in the aliphatic proton range, and the decay was processed with 2D Fourier transform.

### 2.7 CD

Circular dichroism (CD) measurements were performed on a Jasco J-815 CD spectrometer equipped with a thermostable cell holder. Temperature was controlled and monitored with a Peltier type Jasco CDF-426 L thermostat unit. The substrate and different stressed enzyme mixtures were measured together for kinetic studies, to determine the reduction of activity in time. The possible manifestation in secondary structure changes due to the supposed temperature induced degradation was also intended for analyzation. Conformational changes in the secondary structure of protein were monitored in the region of 190–250 nm with a protein concentration of 50 μg/mL (0.60 μmol) in a quartz cuvette (Hellma) with a path length of 1 mm. The scanning speed, bandwidth and data pitch were set to 50 nm/min, 1 nm and 0.5 nm, respectively, while the number of accumulations for each recorded spectrum was set to 3 (within 600 H T voltage range) and averaged to get the complete spectrum.

The activity measurements were followed by CD spectroscopy alternatively, where the ligand ONPG hydrolysis could be measured selectively online, and the kinetic curve could be acquired directly. Kinetic measurements were performed as follows: ONPG (0.050 g / 10 mL) +1 μL enzyme stock solution (10 mg / mL) dissolved in 3 mL of McIlvaine citrate-Na_2_HPO_4_ buffer, pH = 4.6, temperature 37 °C ± 0.05. Detection wavelength was set at 356 nm (ONPG CD maximum).

### 2.8 Peptide and N-glycosylation analysis

For the sequence and glycan identification bottom-up analysis was used. Five different parallel stressed samples were identified to inspect the permanence level of the changes. All reagents were used in relevant volume, comparable to the protein level. The samples corresponding to 100 μg of protein were solved in MilliQ water. For the S-S bridge reduction ammonium bicarbonate buffer at pH 8.0 containing 200 mM of DTT and Rapigest was added. The samples were successively incubated at 60 °C for 30 min, and then cooled at room temperature. Alkylation of cysteine residues by adding 200 mM IAA in 100 mM ammonium bicarbonate was carried out in the dark at room temperature for 30 min. The samples were left to be digested for 90 min at 37 °C by adding trypsin 1:50 w/w ratio considering the total protein content. Lastly, samples were acidified with cc. formic acid to stop the digestion. In the end the samples were centrifuged at 13447 G-force for 10 min and the phases were carefully separated. The trypsin digestion was carried out in solution without TFA in order to avoid the MS source contamination. The mass spectrometric measurements started right after the preparation as follows: 6 μL sample was subjected to nanoLC-MS/MS analysis using a Dionex Ultimate 3000 RSLC nanoLC (Dionex, Sunnyvale, CA, USA) coupled to a Bruker Maxis II Q-TOF (Bruker Daltonics, Bremen, Germany) via CaptiveSpray nanoBooster ion source. Peptides were separated on an Acquity M-Class BEH130 C18 analytical column (1.7 μm, 75 μm × 250 mm Waters, Milford, MA) using gradient elution (4–50 % eluent B in 120 min) following trapping on an Acclaim PepMap100 C18, 5 μm, 100 μm × 20 mm (Thermo Fisher Scientific, Waltham, MA) trap column. Solvent A consisted of water containing 0.1 % formic acid, while Solvent B was acetonitrile containing 0.1 % formic acid. Spectra were collected using a fix cycle time of 2.5 s and the following scan speeds: MS spectra at 3 Hz, while CID was performed on multiply charged precursors at 16 Hz for abundant ones and at 4 Hz for the ones with low abundance. Internal calibration was performed by Compass Data Analysis software 4.3 (Bruker Daltonics, Bremen, Germany) based on infusing sodium-formate. Data were processed by the Byonic software (Protein Metrics, Cupertino, CA, USA). Peptides were identified by searching against the Aspergillus Oryzae (W5ZSH9_ASPOZ) in the UNIprot database. Glycans were quantified with the GlycoPattern software [27].

## 3 Results and discussion

The enzyme activity and various structural aspects of the lactase sample stored under various conditions (Table 1) has been studied in detail. No observable change was found among the control and the frozen samples, so these will not be further discussed. As expected, the largest and most significant changes in enzyme activity and property was observed at the highest temperatures and longest periods. Structural changes corresponding to the observed change in enzyme activity have been studied by a number of analytical methods. In the following discussion structure of lactase, and in particular changes in its structure have been evaluated using a number of analytical methods. Changes in the molecular mass has been studied by SDS-PAGE and NMR. Changes in the molecular size and polydispersity were studied using DLS. Information about the structure changes were obtained by MS and CD spectrometry.

These results will be presented below in detail.

### 3.1 Activity

**Fig. 1:**
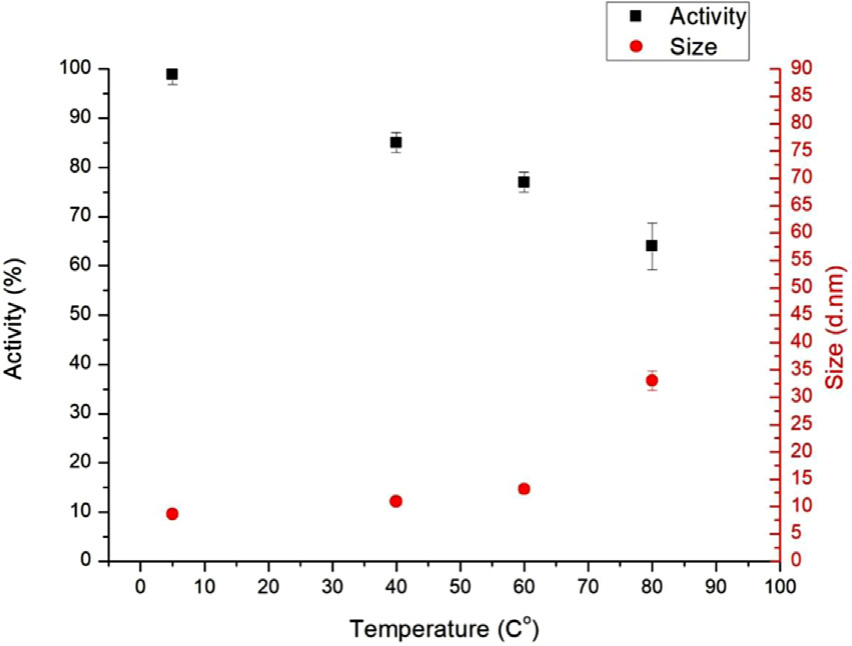
The effect of temperature of heat stress on the β-galactosidase activity (n = 3) and the average hydrolytic diameter (n = 4) of the particles in the standard and different temperature stressed enzyme solutions, immediately after solvation.

**Fig. 2:**
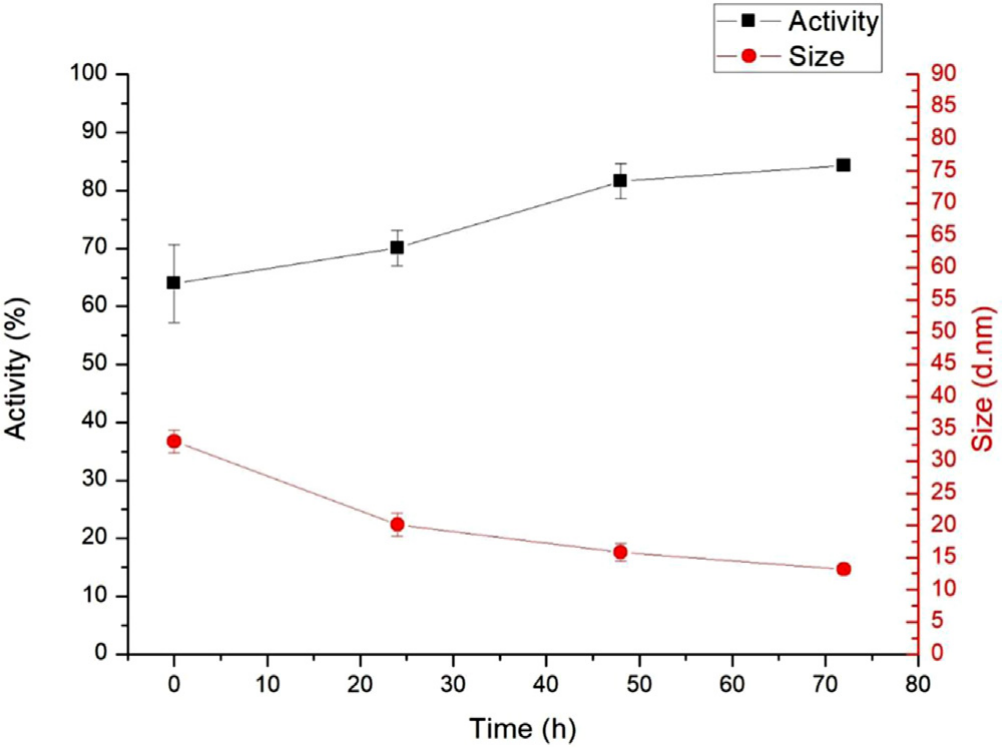
Changes in activity (n = 3) and average molecular size (n = 4) over time in the 80 °C stressed lactase sample in solution.

The changes in lactase activity due to thermal stress are shown in Fig. 1. This clearly shows that enzyme activity is considerably reduced under heat stress. Degradation of the stressed enzyme samples was calculated compared to the activity of the standard sample taken as 100 %. Enzyme activity of the sample stored at 80 °C for one week decreased to 64 % that of the control sample. This is, however, a smaller decrease than occurs is solution (in which case activity decreased to ca. 40 % that of the control sample) [7,28]. It is important, that after dissolving the heat-stressed sample, lactase activity slowly regenerates in time (in hours or days, from 64 to 80 % that of the original activity). This is shown in Fig. 2. This suggests changes in the sample due to heat-stress are partially reversible.

### 3.2 DLS

**Fig. 3:**
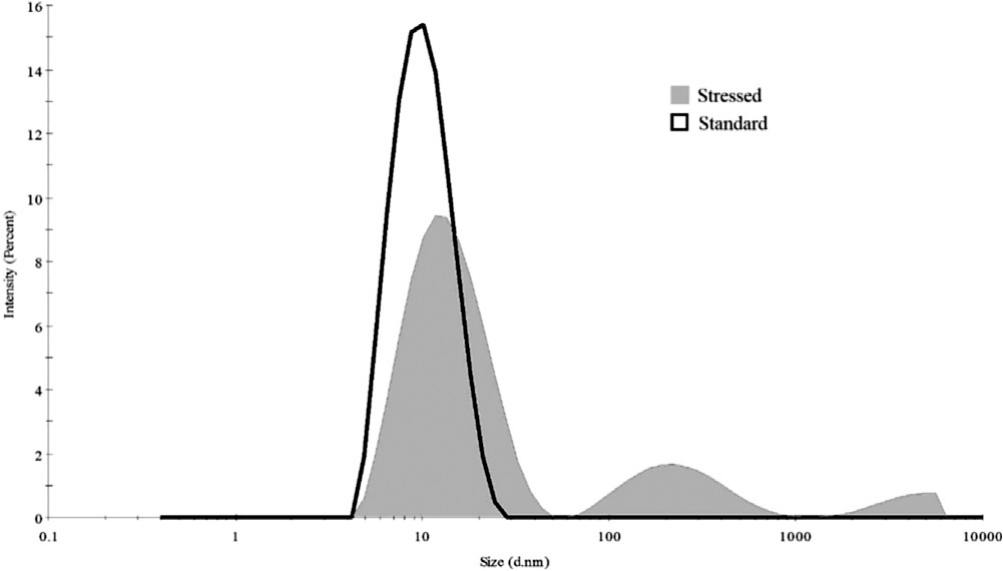
Size distribution of the standard and heat stressed protein determined by DLS measurement.

Having observed changes in lactase activity due to heat-stress, we have tried to understand the corresponding changes in protein structure. First, we have performed dynamic light scattering (DLS) measurements to characterize molecular size and possible aggregation. We have determined the average molecular size (hydrolytic diameter using a hard sphere model) and the polydispersity index. Note, polydispersity indicates the variability of the molecular size based on the DLS distribution [29,30]. The DLS distribution is shown in Fig. 3, both that of the standard and the heat-stressed sample. The hydrolytic diameter of the particles in the standard enzyme solution was between 9 and 10 nm in each measurement, and polydispersity index was less than 0.2, suggesting a narrow distribution. These indicate, that under normal conditions the enzyme in solution is in the form of monomers. Fig. 1 shows that heat stress (which decreases enzyme activity) increases the average particle size, indicating aggregation and protein unfolding. Dissolving the heat-stressed sample, it was shown that enzyme activity slowly recovers (Fig. 2). In parallel, particle size decreases, indicating that aggregation (and/or unfolding) is partially reversible.

Increase of particle size is small at low temperatures and is likely due to protein unfolding. At higher temperatures (80 °C) large, 2−3-fold increase in average particle size can be observed. Fig. 3 shows that in this case as large as micrometer sized particles also appear, clearly indicating protein aggregation. This figure also shows that the first peak in the particle distribution of the heat-stressed sample is wider than that of the standard sample. This smaller change in particle size suggests protein unfolding.

### 3.3 SDS-PAGE

**Fig. 4:**
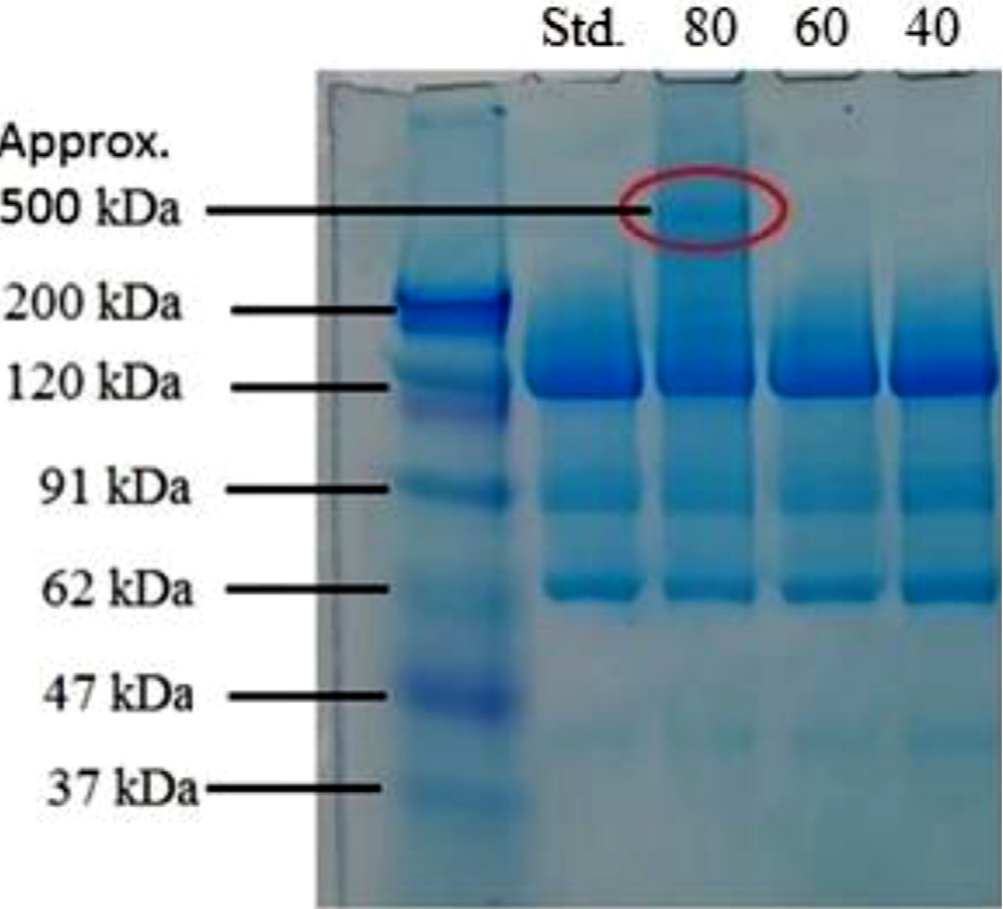
SDS-PAGE electropherogram of the Standard and heat stressed enzymes. The contents of the lanes from left to right are the following: Lane 1: molecular mass markers, mass indicated alongside. Lane 2: Standard β-gal. enzyme. Lane 3-5: Heat stress.

According to the information in the UNIprot database [6], the weight of β-galactosidase from Aspergillus oryzae is 109 kDa. This information is based on the amino acid sequence and does not take into account possible structural modifications. Our preliminary MALDI-TOF measurement indicates a molecular mass of 123 kDa, which suggests extensive post-translational modifications (PTMs). We have studied changes in the molecular mass of the control sample and heat stressed samples stored at 40, 60 and 80 °C for one week using SDS-PAGE. The result is shown in Fig. 4.

In the first column a standard protein ladder was used to determine where the different masses of materials are located on the gel. There is no visible change in molecular weight in the case of 40 °C, 60 °C heat stressed samples, but at 80 °C a band appeared at higher (approximately 500 kDa) molecular weight, from which it can be concluded that the protein starts to aggregate and likely forms tri- or tetrameric structures, as suggested [31]. It can also be seen on the gel that the loaded sample drew a continuous strip, presumably due to the presence of larger but not well-defined particles in comparison to the main component. Both the standard and stressed sample contain molecules with lower molecular mass at 90, 60 and 40 kDa (Fig. 4). The presence of these bands suggests contaminants or degradation products. As the presence of these bands did not change due to heat stress, these were not further studied.

### 3.4 NMR

The NMR measurements provided information on the structure and size of the protein. The large molecular size and heterogeneous composition of the lactase samples caused some practical difficulties regarding NMR measurements. For this reason, the classical Stejksal-Tanner equation fitting did not give useful results, as regression always resulted in a multicomponent fitting. Information on various size molecular components in the lactase sample (i.e. small molecules, intact lactase and lactase aggregates were obtained using 2D DOSY spectroscopy). This revealed that multiple diffusion constants can be detected in the samples with differences both in size and distribution. The specified diffusion constants and the calculated masses are extrapolated (based on the work of Dudás and Bodor [26]) are summarized in Table 2.

**Table 2:** The diffusion constant values of the different particles in the standard and 80 °C stressed samples and the equivalent masses.

Lactase standard:	Stressed sample (80 °C):
6.47^•^10^−11^ m^2^s^−1^ (27 kDa)	N/A
4.86·10^−11^ m^2^s^−1^ (58 kDa)	4.88·10^−11^ m^2^s^−1^ (57 kDa)
3.63·10^−11^ m^2^s^−1^ (124 kDa)	3.66·10^−11^ m^2^s^−1^ (121 kDa)
N/A	2,75 · 10^−11^ m^2^s^−1^ (257 kDa)
N/A	2.75·10^−11^ m^2^s^−1^ (550 kDa)

The 27 and 58 kDa size ranges in the measurements are not caused by the thermal stress; they are present in both samples from the beginning like presented earlier in Fig. 4. These are likely to be contaminants or degradation products in the commercial lactase sample. The particles observed following heat stress are likely di- and tetramer aggregates of the lactase molecule (257 and 550 kDa).

**Fig. 5:**
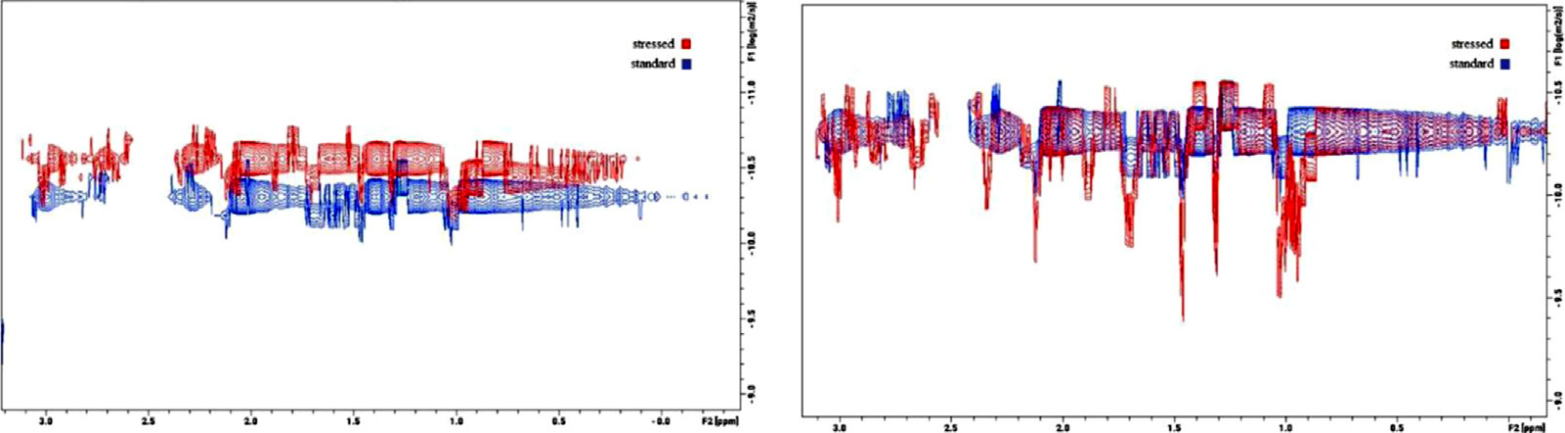
The 2D DOSY 1H NMR spectra of the same sample detected 2 h (left) and 72 h (right) after dissolving the solid samples in water. The DOSY profile belonging to the stressed sample (red) return to the standard’s profile (blue) after 72 h.

Both the DOSY and ^1^H-NMR patterns change over time in the samples. Based on the DOSY spectra, the samples were found to be mixtures characterized by multiple diffusion constants. The molecule sizes determined based on DOSY are significantly larger in the stressed samples (Table 2). The dissolved protein aggregates are unstable among these circumstances in accordance with the former observations. A definite alteration in DOSY pattern could be observed over time in case of the heat stressed samples dissolved for NMR measurement. Interestingly, the fraction of DOSY signals corresponding to the protein aggregates decreases over time, and after 72 h the DOSY pattern of the heated samples finally reaches that of the untreated sample. This indicates that aggregation occurs in the solid state during heat stress, but it is mostly reversible in solution (Fig. 5).

**Fig. 6:**
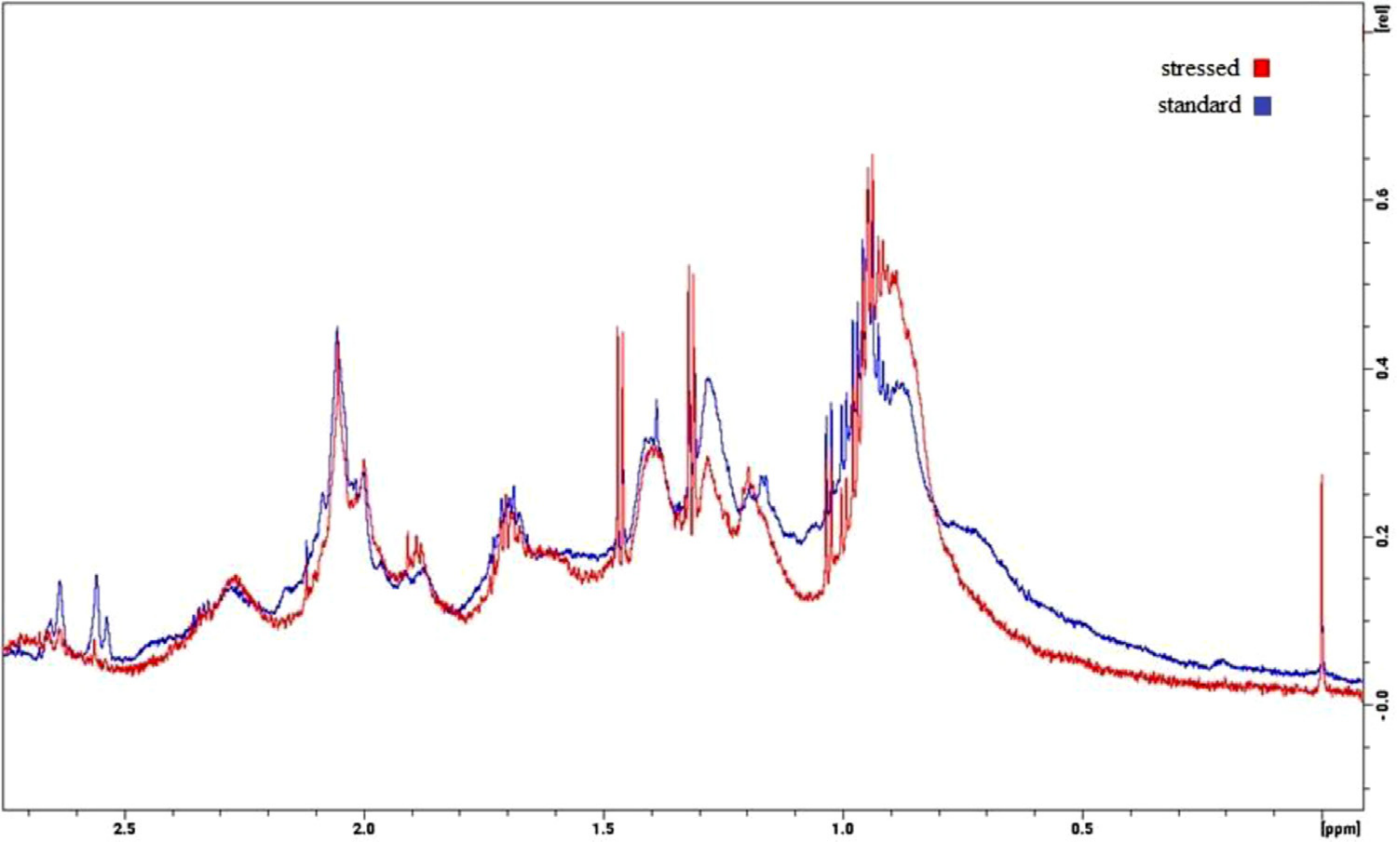
The ^1^H spectrum of the standard and stressed samples.

The intensity of sharp signals of small molecules is significantly growing in the ^1^H spectra of the heated samples (Fig. 6). The increase in the intensity of the small molecule signals in the ^1^H-NMR spectra is presumably associated with the small carbohydrate molecules arising due to the degradation of the glycan chains from the protein. Glycan degradation has been confirmed by the MS study, and will be discussed below in detail.

### 3.5 Circular dichroism

**Fig. 7:**
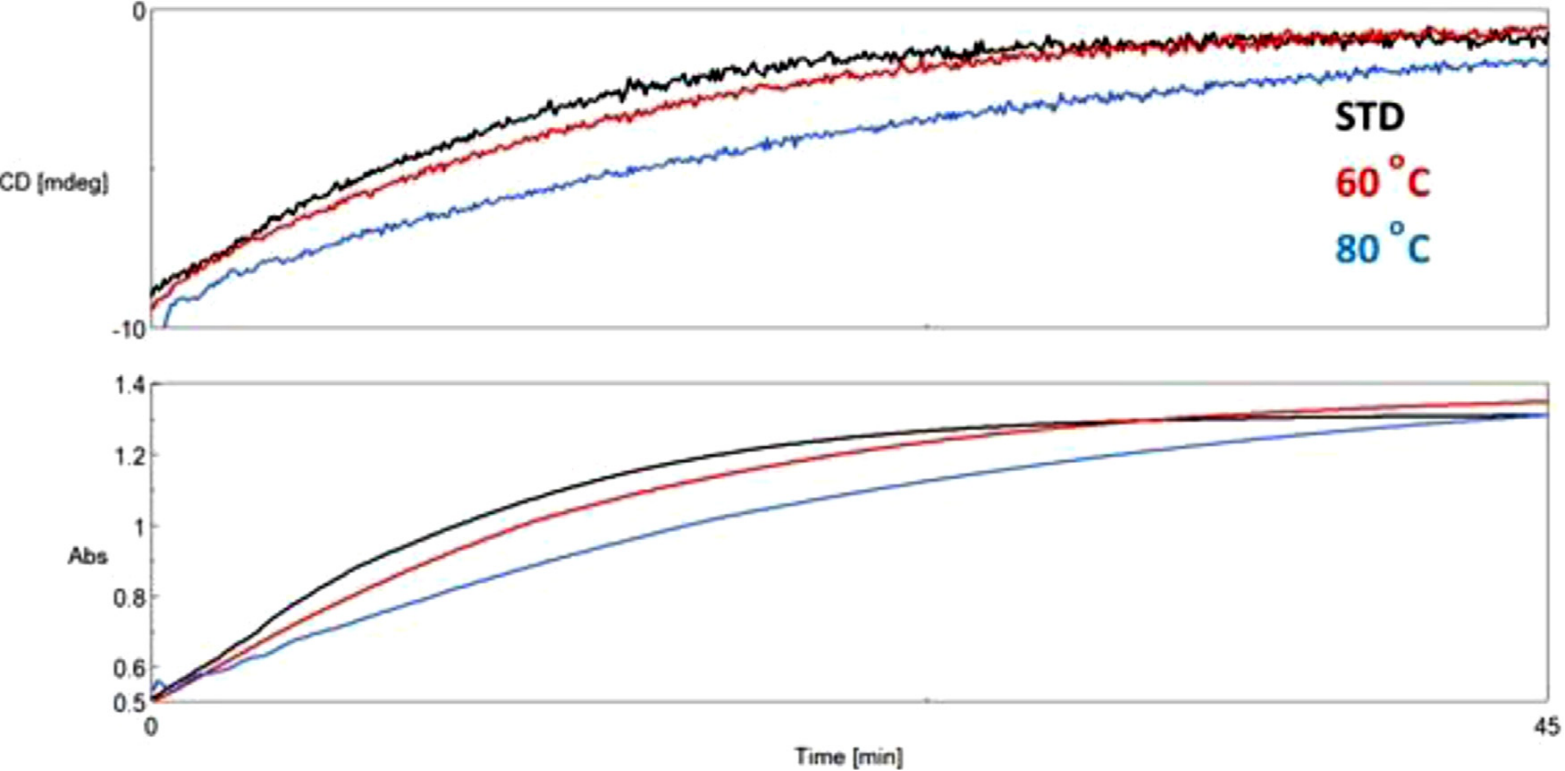
Lactose and ONPG CD (Upper) and UV (Bottom) kinetic curves of same concentration at 37 °C.

Lactase activity changes were observed/confirmed by CD kinetic studies, where enzyme kinetics was studied by observing both lactase and its substrate. Direct monitoring of the reaction was accomplished by dual CD / UV detection. It was possible, as the amount of substrates, which formed as a result of the catalytic reaction of ONPG can be selectively measured in the solution, because galactose is chiral and the ortho-nitro phenol is a good chromophore. During the hydrolysis, the disruption of the glycosidic bond causes the disappearance of CD signal and the strengthening of UV signal. The CD curve selectively measures the hydrolysing substrate, while the increase in the UV curve indicates the formation of dinitrophenol (Fig. 7). Results obtained from enzyme kinetic CD studies correlate well with activity measurements (Fig. 1).

**Fig. 8:**
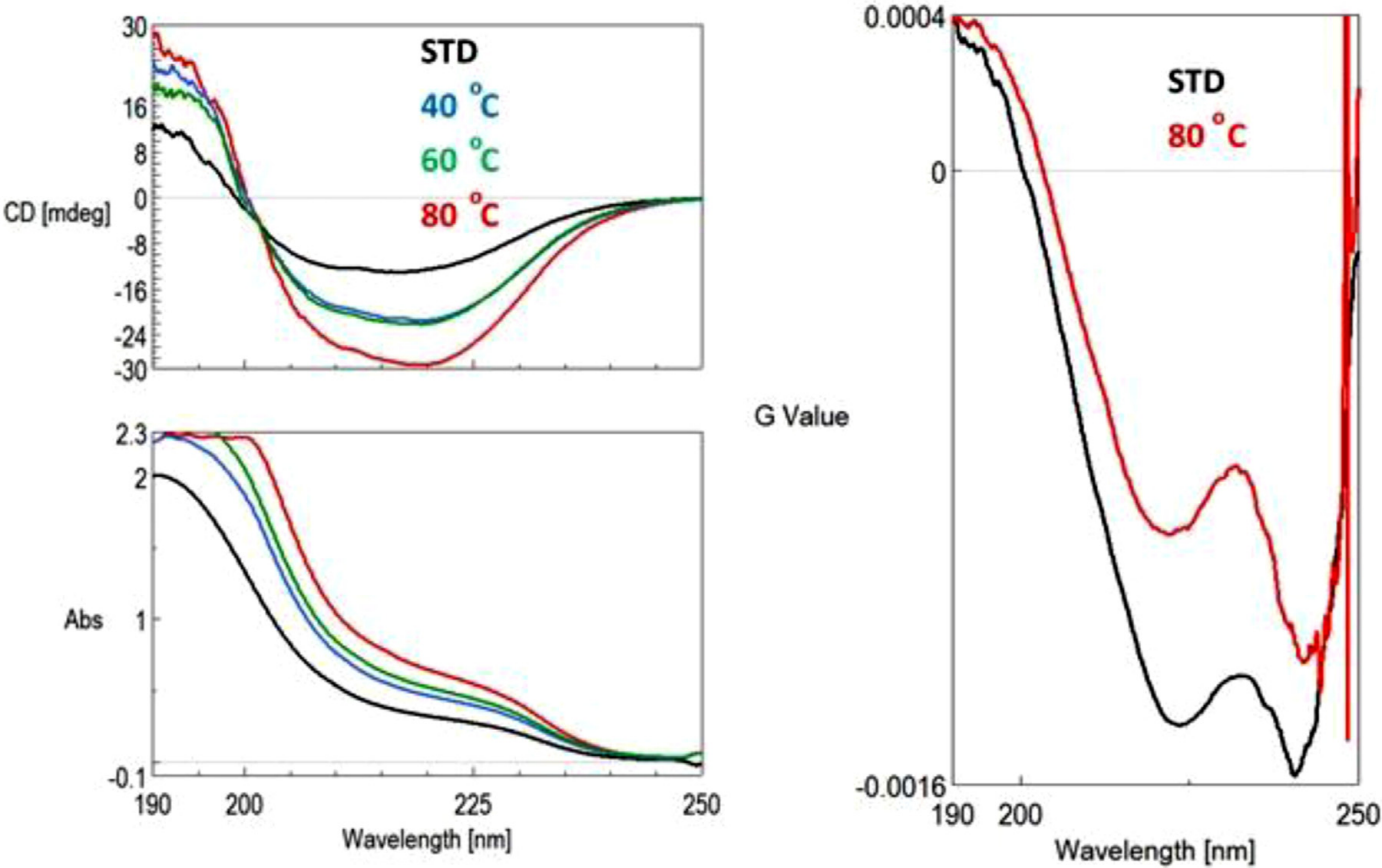
CD and UV spectra of freshly prepared lactase samples (left) and g-spectra of lactase standard and lactase stored at 80 °C (right).

The possible conformational changes in the secondary structure were also analysed. The differences between the spectra may be caused by the different initial concentrations, but if we calculate the Dissymmetry Factor (g), which is the ratio of the CD and the UV signals and a dimensionless parameter, then the concentration differences disappear. Furthermore, if there is a slight but significant difference in either the CD or the UV spectrum, it appears on the g-spectrum [32]. The following Fig. 8 shows the CD and UV spectra of samples on the left and the g-spectra of standard and treated lactase at 80 °C on the right side. The intensities differ due to the different concentrations. However, the value 'g' (CD / UV ratio spectrum), which is independent of concentration, does not differ significantly in the case of the standard and the samples stored at 40 and 60 °C, which means the residual protein still does not lose its structure. A change in the intensity of g- spectrum indicates some structural differences comparing the standard and the treated sample at 80 °C. Real evidence for the structural change is the ratio of the peaks at 241/222.4 nm, where the value is 1.086 for the standard and 1.307 for the treated sample.

### 3.6 Peptide and N-glycosylation analysis

**Fig. 9:**
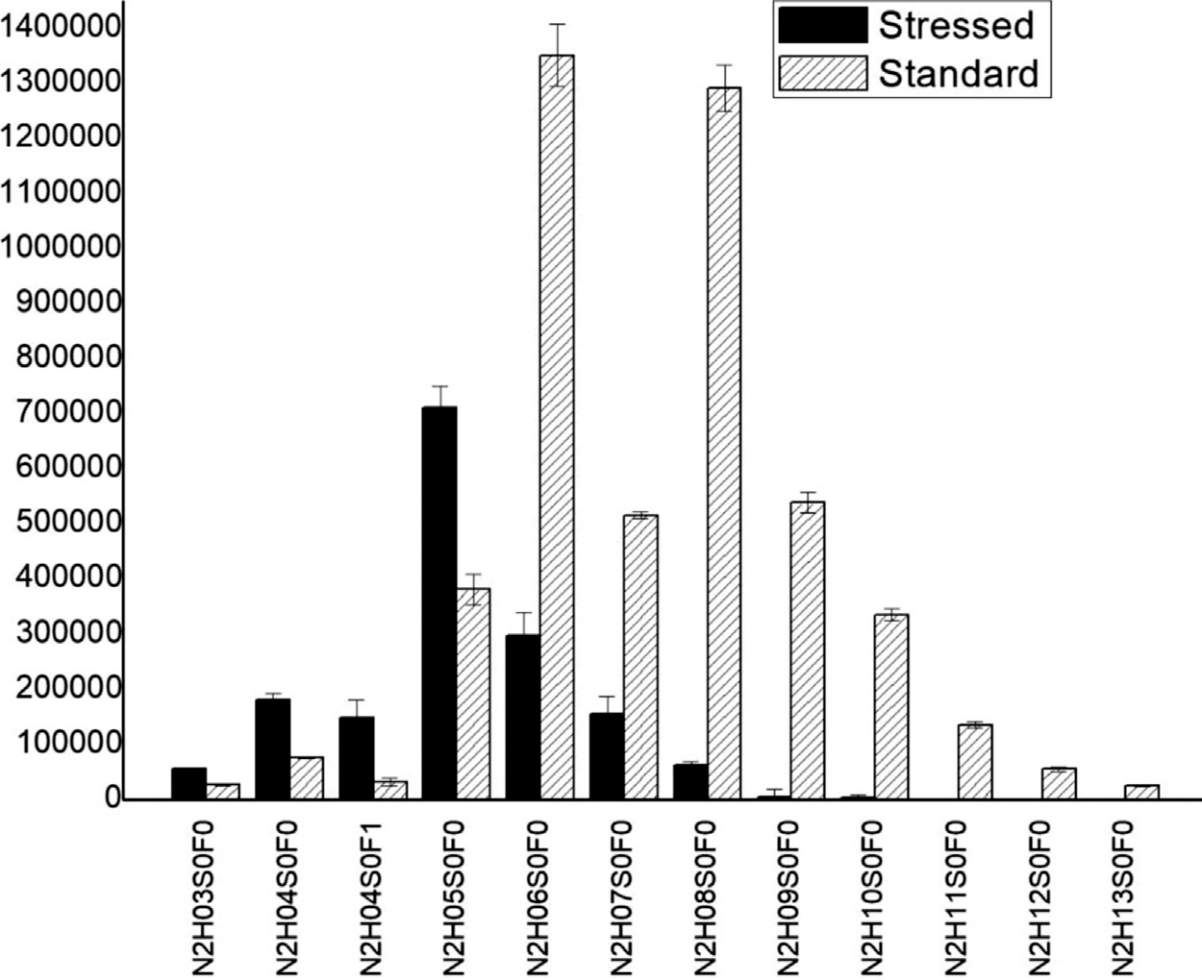
Section of the glycosylation pattern of NLTTGVYTDTSDLAVTPLMGDSPGSFFVVR, comparing the average abundance of the glycans of the stressed and standard samples on the NLTTGVYTDTSDLAVTPLMGDSPGSFFVVR glycopeptide. (N: N-acetylhexosamine, H: hexose, S: sialic acid, F: fucose).

**Fig. 10:**
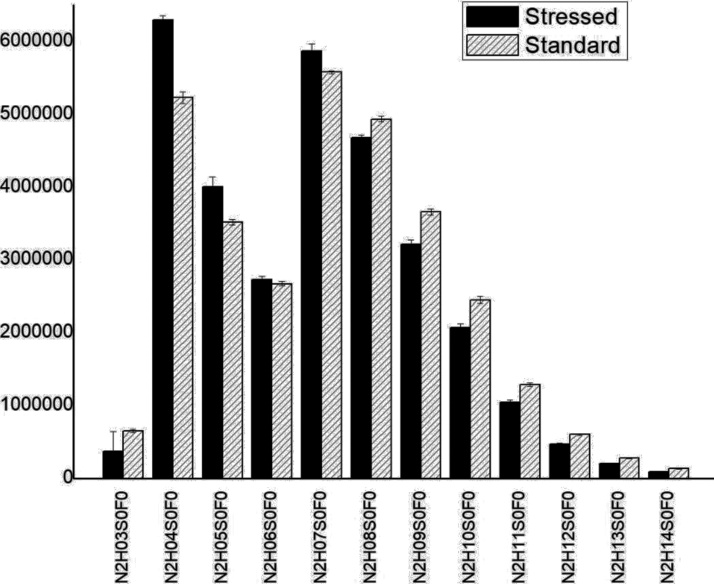
Section of the glycosylation pattern of VNGLTR, comparing the average abundance of the glycans of the stressed and standard samples on the VNGLTR glycopeptide. (N: N-acetylhexosamine, H: hexose, S: sialic acid, F: fucose).

The lactase enzymes (the control and heat-stressed samples) were studied, after tryptic digestion, using LC–MS/MS analysis. Peptides were identified using the Byonic software. Very high, 95.02 % sequence coverage was observed for the control sample, while 91.4 % for the 80 °C stressed sample. Recent studies on other proteins [9,33] suggest glycans has a large inflence on enzyme activity. For this reason, we have studied N-glycosylation of lactase in detail, for which very little information is available. We have found 8 different glycosylation sites; each was substituted by a large number of glycans (Table 3). The glycans contained N-acetylhexosamine, hexose, sialic acid and fucose residues, and most had a high-mannose structure (Fig. 9, Fig. 10). Note, our LC–MS analysis allowed site-specific glycosylation analysis (i.e. to have information on the glycans observed at the various glycosylation sites). Most glycans were so-called complex and high mannose glycans.

**Table 3:** The table shows the examined peptides, the number of found glycans, the number of statistically significant differences between the samples.

Peptide	Number of found glycans	Significant Differences
VNGTLR	25	21
SNVTIIEGSDSGIVSTR	26	20
GWDVPLYFNFGNNTQAAR	13	5
LPTSAGNLTIPQLEGTLSLNGR	24	17
LKLPTSAGNLTIPQLEGTLSLNGR	20	12
IHVVDYNVSGTNIIYSTAEVFTWK	19	19
NLTTGVYTDTSDLAVTPLMGDSPGSFFVVR	16	16
GAHLDGADLHLTADFNATTPIEVIAPTGAK	5	5

Heat-stress caused a large (and irreversible) change in glycosylation. This was revealed by quantitative mass spectrometric analysis. For quantitative comparison 5 replicates of the control sample, and 5 replicates of the 80 °C heat stressed sample were analysed. The glycopeptides found in both samples were selected for further investigation. A *t*-test was used to identify those glycans, that showed significant (p = 0.05) difference between the control and the heat-stressed sample (Table 3). The glycosylation pattern (relative glycoform abundances) for two glycosylation sites are shown in Fig. 9, Fig. 10. The glycopeptide in Fig. 9 (NLTTGVYTDTSDLAVTPLMGDSPGSFFVVR) shows a very large change due to heat-stress: The largest change occurred in glycan size, in particular the number of hexose units present. Due to heat-stress the average number of hexose units decreased form ca. 8 to 5. In the other example (VNGLTR) the change in glycan composition was not so large, but still observable, and statistically significant (Fig. 10 and Table 3). Note that the buildup of complex glycans occurs in living cells, so their degradation is irreversible.

## 4 Conclusion

Our investigations indicate various changes occur when submitting the solid enzyme to heat stress. Heat stress causes significant decrease in enzyme activity. However, after dissolution, in several hours, enzyme activity somewhat recovers, suggesting the activity decrease is partly reversible. This can be explained by reversible aggregation of lactase molecules, suggested by the results of DLS and NMR measurements. In the case of oral medication, partial recovery of enzyme activity is not relevant, as lactase molecules do not spend sufficient time in dissolved form in the body. The amino acid sequence and the secondary structure of the enzyme did not show any change due to heat stress. Lactase is heavily glycosylated, and heat stress did change glycosylation significantly. LC–MS experiments revealed that various peptide-bound glycan chains were shortened due to heat stress. These are irreversible changes, changeing the physico-chemical properties of the enzyme and may increase tendency of aggregation.

These studies suggest that both structural changes in the higher order structure of the protein and changes due to altered glycosylation are responsible for the decrease of activity. Understanding behaviour under heat stress may help to design better manufacturing, shipping, and storage conditions and improve our understanding of different types of lactases, like Kluyveromyces lactis used in dairy industry or even human lactase. The methodology developed here may serve as a model for investigating other enzymes of pharmaceutical interest.

## CRediT authorship contribution statement

**Márton Király:** Writing - original draft, Visualization, Formal analysis, Investigation, Methodology. **Borbála Dalmadi Kiss:** Writing - review & editing, Methodology, Conceptualization. **Péter Horváth:** Investigation, Writing - original draft, Visualization. **László Drahos:** Software, Formal analysis, Writing - review & editing. **Arash Mirzahosseini:** Funding acquisition, Writing - original draft. **Gyula Pálfy:** Investigation, Visualization, Writing - review & editing. **István Antal:** Resources. **Krisztina Ludányi:** Conceptualization, Supervision, Funding acquisition, Writing - review & editing, Methodology.

## Declaration of Competing Interest

The authors declare that they have no known competing financial interests or personal relationships that could have appeared to influence the work reported in this paper.

## Appendix A Supplementary data

Supplementary data contains the evaluated mass spectrometry results sorted according to the glycopeptides we examined. The data displays the aboundance of the selected glycopeptides and the figures show the results of the parallel measurements and their average.
